# Transcription cofactor Hes6 interacts with Twist1 to facilitate EMT and promote gastric carcinogenesis by activating the PI3K/AKT signaling

**DOI:** 10.1016/j.gendis.2025.101674

**Published:** 2025-05-05

**Authors:** Can Fang, Zhiwei Peng, Yaru Sang, Zihao Ren, Kang Wang, Nuo Xu, Ying Li, Tingting Guo, Yinan Zhu, Shangxue Yan, Kongwang Hu

**Affiliations:** a Department of General Surgery, The First Affiliated Hospital of Anhui Medical University, Hefei, Anhui 230032, China; b Department of General Surgery, Fuyang Hospital of Anhui Medical University, Fuyang, Anhui 236000, China; c Institute of Clinical Pharmacology, Anhui Medical University, Key Laboratory of Anti-inflammatory and Immune Medicine, Ministry of Education, Hefei, Anhui 230032, China; d Laboratory Animal Center, Anhui Medical University, Hefei, Anhui 230032, China; e Department of Hepatobiliary and Pancreatic Surgery, General Surgery Center, The First Hospital of Jilin University, Changchun, Jilin 130021, China; f Department of Otolaryngology Head and Neck Surgery, Nantong First People's Hospital, Nantong, Jiangsu 226001, China

**Keywords:** EMT, Gastric cancer, Hes6, PI3K/AKT pathway, Twist1

## Abstract

Advanced gastric cancer (GC) poses a significant threat to public health, leading to substantial consumption of healthcare resources due to its aggressive nature and poor prognosis. Hairy and enhancer of split 6 (Hes6), a member of the mammalian homologs of *Drosophila*'s hairy and enhancer of split (HES) family, functions as a transcriptional cofactor owing to its unique structural features. Previous studies have indicated that Hes6 expression is elevated in several malignant tumors, contributing to the enhanced proliferation and invasion of tumor cells. However, the precise role and underlying mechanisms of Hes6 in gastric cancer initiation and progression remain poorly understood. In this study, we demonstrate that Hes6 expression is significantly up-regulated in gastric cancer tissues, with elevated levels of Hes6 correlating with poor prognosis in certain patient cohorts. Functionally, Hes6 mediates gastric cancer cells to obtain stronger proliferation, migration, and invasion abilities by activating the PI3K/AKT signaling pathway, thereby accelerating tumor progression. Moreover, Hes6 interacts with the Twist1 protein, stabilizing it and facilitating the epithelial-mesenchymal transition (EMT). Collectively, these findings offer valuable insights into the regulatory mechanisms underlying gastric cancer progression, highlighting the potential of transcriptional cofactors, such as Hes6, in PI3K/AKT signaling as promising targets for therapeutic intervention in gastric cancer.

## Introduction

Gastric cancer (GC) is a particularly aggressive and heterogeneous malignancy, and it ranks among the leading causes of cancer-related morbidity and mortality globally.^1^^,^^2^ Metastasis manifests in roughly 40% of GC patients, with a considerable proportion of newly diagnosed cases confronting regional metastases inevitably. Meanwhile, individuals with metastatic diseases typically have a five-year overall survival rate of approximately 5%.^1^^,^^3^ The intricate interaction of environmental and genetic variables contributes to the prominent heterogeneity and complexity of GC, resulting in relative ineffectiveness of surveillance and treatment strategies.^3^^,^^4^ Management of patients with this disease remains a significant challenge. Urgent exploration into the broader and detailed molecular mechanisms underlying GC development, as well as the formulation of innovative therapeutic options, is essential.

Hes6 is a member of the mammalian homologs of *Drosophila's* hairy and enhancer of split (HES) family. Unlike other HES family members, the loop region of Hes6's basic helix-loop-helix (bHLH) domain is shortened by four or five amino acid residues, which impairs its ability to bind directly to DNA through this domain. As a result, Hes6 is classified as a transcriptional cofactor rather than a direct DNA-binding transcription factor.^5^ The interactions with other DNA-binding proteins facilitate its recruitment to DNA to exert its biological functions.^6^^,^^7^ In terms of mechanism, transcription cofactors serve as important components in eukaryotic cells, work synergistically with transcription factors to form transcription complexes, and directly bind to regulatory elements in an interdependent manner.^8^ Transcription cofactors have a key influence on transcription factor activity, which provides precise regulation of the expression of specific genes and controls the transcriptional network inside cells, thereby maintaining the homeostasis of a variety of complex biological metabolic processes.^9^ For example, recent research has reported that Hes6, as a critical transcriptional cofactor, mediates human erythropoiesis by forming a regulatory loop with GATA-binding factor 1 (GATA1).^10^ Interestingly, Hes6 is indeed up-regulated in a variety of solid tumors,^11^^,^^12^ indicating its potential involvement in multiple cancer-related functions. Despite the several studies that have explored the role of Hes6 in cancer, there remain insufficient insights that are comprehensive and in-depth. A recent study utilizing single-cell RNA sequencing technology identified Hes6 as a potential marker for pre-goblet cell clusters, with the appearance of goblet cells being generally considered to be an early hallmark event that may progress to gastric adenocarcinoma.^13^ Nevertheless, there have been few studies investigating the role and molecular mechanisms of Hes6 in GC.

In this study, we set out to explore the expression of Hes6 in GC and its clinical relevance and found that it was overexpressed in GC tissues. Subsequently, with *in vivo* and *in vitro* functional assays, it was observed that overexpressing Hes6 could enhance proliferation and metastatic potential in GC cells, while knocking down Hes6 could diminish the malignant traits of these cells. In addition, we further examined the underlying mechanism by which Hes6 promoted cancer progression, and the Twist1 protein was screened and verified as an interacting molecule that Hes6 can bind to Twist1 as a transcriptional cofactor, enhancing its stability and facilitating the epithelial-mesenchymal transition (EMT) process. At the same time, we determined that the phosphoinositide 3-kinase (PI3K)/protein kinase B (AKT) signaling pathway coordinated the function of Hes6 in regulating the malignant behavior of GC cells. In conclusion, our findings together elaborated the distinct role and mechanism of Hes6 in GC and presented a potential therapeutic target to some extent.

## Material and methods

### Clinical samples and cell lines

The human specimens (20 cases) were collected from GC patients who underwent surgical procedures at the First Affiliated Hospital of Anhui Medical University (Hefei, China) between March and April 2023. The detailed information of the samples is shown in Table S1. All study protocols adhered to the guidelines set forth by the regional Committees for Ethical Review of Research and were conducted in accordance with the 1964 Helsinki Declaration. Informed consent was obtained from all participants before their involvement in the study and before the surgical procedures. The GC cell lines utilized in this study were acquired from Shanghai Guandao Bioengineering Co., Ltd. (Shanghai, China). These cells were cultured in RPMI 1640 medium, supplemented with 10% fetal bovine serum (Wisent, China) and 1% penicillin-streptomycin (Beyotime, China), and maintained in a humidified incubator with 5% CO_2_ at 37 °C. All cell lines were examined regularly to prevent mycoplasma contamination.

### Total RNA extraction and real-time quantitative PCR

Total RNA from frozen GC tissues (about 20 mg per sample) or cultured cells (about 5–8 × 10^5^) was extracted using TRIzol reagent (Invitrogen, Carlsbad, CA, USA), according to the manufacturer's instructions. The RNA concentration and purity were immediately assessed using a NanoDrop 2000 spectrophotometer (Thermo Fisher Scientific, USA). A total of 1000 ng of RNA was reverse-transcribed into cDNA using the Hiscript®Ⅱ 1st Strand cDNA Synthesis Kit R 211 (Vazyme, Nanjing, China), following the provided protocol. Real-time quantitative PCR (RT-qPCR) was conducted using the Quant Studio 3 Real-Time PCR System (Thermo Fisher Scientific, USA) with ChamQ Universal SYBR qPCR Master Mix (Vazyme, Nanjing, China). GAPDH or β-actin served as the internal control. The primers for RT-qPCR are detailed in Table S2.

### Lentivirus infection

To construct GC cell lines that stably overexpress or knock down Hes6, AGS, SGC 7901, and HGC 27 cells were transfected with lentiviral containing small-hairpin shRNAs and overexpression vectors for Hes6, as well as a negative control. The detailed information about shRNAs and plasmids is exhibited in Table S2. After transfection, the cells were selected using puromycin (1–2 μg/mL) for at least two weeks. All lentiviral vectors were designed and synthesized by Genechem Co., Ltd., Shanghai, China.

### Immunohistochemistry

The detailed experimental procedures of hematoxylin-eosin staining and immunohistochemistry assay covered in our study are consistent with what we reported previously.^14^^,^^15^ The primary antibodies used are listed below: Ki-67 (1:200, HuaBio, HA721115). After panoramic scanning of each immunohistochemistry section, a fixed range of fields of view was randomly selected as representative images.

### Western blotting analysis

Cells (about 5–8 × 10^5^) and GC samples (about 30 mg per sample) were harvested and lysed on ice using RIPA lysis buffer (PC101, Epizyme, China) supplemented with protease inhibitor (#HY-K0010, MCE, China) and phosphatase inhibitors (#HY-K0021 & #HY-K0022, MCE, China) for 30 min. The lysates were centrifuged at 15000 rpm at 4 °C for 20 min. The supernatants were collected, and protein concentrations were determined using the BCA Protein Assay Kit (P0012, Beyotime, China). The lysates were then mixed with protein loading buffer, and the proteins were denatured by boiling at 100 °C for 8 min. Equal amounts of protein samples were subjected to sodium dodecyl sulfate-polyacrylamide gel electrophoresis (SDS-PAGE), followed by transfer of the proteins onto a polyvinylidene fluoride (PVDF) membrane. The membrane was blocked with 5% skim milk at room temperature for 30 min and then incubated overnight at 4 °C with primary antibodies diluted in primary antibody dilution buffer (Beyotime, China). The primary antibodies used in this study are as follows: β-actin (1:5000, HuaBio, EM21002), Hes6 (1:1000, Immunoway, YT2127), Hes6 (1:1000, Abmart, MG280717), N-cadherin (1:5000, Abclonal, A0433), E-cadherin (1:2000, Proteintech, 20874-1-AP), Vimentin (1:1000, Proteintech, 10366-1-AP), Snail (1:1000, Abmart, TA6032), Twist1 (1:1000, Proteintech, 25465-1-AP), PI3K (110α) (1:1000, CST, 4249S), PI3K (85α) (1:1000, Proteintech, 60225-1-lg), Phospho-PI3K p85 (Tyr458) (1:1000, Affinity, AF3242), Phospho-Akt (Ser473) (1:1000, CST, 4060T), and AKT (1:1000, CST, 4691T). The membranes were washed with Tris-buffered saline containing 0.1% Tween 20 and subsequently incubated with HRP-conjugated secondary antibodies, namely, goat anti-mouse (catalog no. SA00001-1, 1:10000, Proteintech) or goat anti-rabbit (catalog no. SA00001-2, 1:10000, Proteintech), at room temperature for 2 h. Finally, protein bands were visualized with the ChemiDoc Imaging System (Bio-Rad, USA) using an ECL substrate kit (P10060, NCM Biotech, Suzhou, China).

### Cell proliferation assay

The AGS, HGC 27, and SGC 7901 cells, which were infected with lentivirus, were seeded into a 96-well plate at a density of 3000 cells per well. Cell viability was assessed using the Cell Counting Kit-8 (Biosharp, China) following the manufacturer's instructions to evaluate cell proliferation.

### EdU assay

Stably transfected cells were plated at a density of 5 × 10^4^ cells per well in 24-well plates and incubated for 24 h before performing the experiment using the EdU assay kit (#C0075S, Beyotime Biotechnology, Shanghai, China) following the manufacturer's protocol. Images were captured using a fluorescence microscope (Leica DM4B, Germany) (more detailed parameter information is listed in Table S3). The percentage of EdU-positive cells was calculated using ImageJ software according to the formula: percentage of EdU-positive cells = number of EdU-positive cells/total number of cells × 100%.

### Wound healing assay

Transfected GC cells were seeded into 6-well plates until the density of cells reached 80%–90%. Straight scratches of equal width were made using a sterile 200 μL pipette tip (0 h), and images were captured using microscopy (Leica DMi1, Germany) immediately. The cells were then cultured in a serum-free medium. After 24 h and 48 h, the original images of the cell wound area were acquired again using microscopy and subsequently subjected to further processing (region annotation) with Photoshop software. The area of the wound region was then quantified using ImageJ software, ultimately enabling the calculation of the relative migration rate.

### Colony formation assay

For colony formation assays, stably transfected GC cells were digested and resuspended at a concentration of 1000 cells/mL. The cell suspension was seeded evenly in 6-well culture plates at a density of 1000 cells per well and cultured at 37 °C in a 5% CO_2_ atmosphere for 10–14 days. The medium was changed twice during this period. Afterward, the colonies were fixed with methanol and stained with a 0.5% crystal violet solution (Beyotime). Images of the colonies were captured using a camera.

### Transwell assay

For transwell migration and invasion assays, a 24-well transwell chamber (Corning Inc., USA) was employed to assess cell migration and invasion abilities, with or without the application of Matrigel (Corning Inc., USA) to the membrane, respectively. The cells were resuspended in 200 μL serum-free medium and added to the upper chamber at a density of 2 × 10^4^ cells per well. Approximately 600 μL of medium containing 20% fetal bovine serum was placed in the lower chamber. After incubation for 24 or 48 h, cells in the upper chamber were gently removed using a cotton swab. Cells that had migrated to the lower membrane surface were then fixed with 4% paraformaldehyde and stained with 0.5% crystal violet for imaging and quantification. The number of migrated and invaded cells was counted in three randomly selected fields.

### Animal experiments

For xenograft proliferation studies, six-week-old male BALB/c nude mice were obtained from GemPharmatech Co., Ltd. (Jiangsu, China). The mice were raised and acclimatized in a specific pathogen-free environment for a period of time before being used in the experiments. The mice were randomly assigned to different experimental groups. SGC 7901 cells (5 × 10^6^ cells/mouse) with stable overexpression/knockdown of Hes6 and the corresponding control group were carefully injected subcutaneously in the right axilla of each mouse. When subcutaneous tumors gradually formed, the tumor growth volume was dynamically recorded. The mice were sacrificed four weeks later, and tumor tissues were harvested. The tumor volume of each mouse was measured and recorded. The tumor volume was calculated using the formula: V (volume, mm^3^) = 0.5 × L (length, mm) × W^2^ (width, mm). The tumor samples were subsequently fixed in 4% paraformaldehyde and embedded in paraffin to prepare FFPE sections. Hematoxylin-eosin staining was performed to examine cell morphology and tissue architecture. Immunohistochemical staining of Ki-67 (1:200, HuaBio, HA721115) was conducted to assess cell proliferation. All animal experiments adhered to ethical guidelines and received approval from the Experimental Animal Ethics Committee of Anhui Medical University.

### Immunoblotting and co-immunoprecipitation assay

First, the cell samples were lysed, and the total protein preparation was extracted. Then, a mixture of Flag/Twist1 antibody (4 μL) and agarose microbeads (20 μL, Santacruz) was added, and IgG was used as a control. The centrifuge tube was carefully vortexed and left to incubate at 4 °C overnight. The following day, the precipitate was spun down at 4 °C and washed with 1 × wash buffer. Subsequently, the protein was eluted from the beads using 1 × SDS loading buffer and heated at 95 °C for 10 min. Lastly, a Western blot assay was conducted to confirm the results.

### Protein half-life assay

Stable overexpressing Hes6 cells were treated with the protein synthesis inhibitor cycloheximide (10 μM; MCE) for specific periods before obtaining total protein, which was then subjected to Western blotting.

### Multiple immunofluorescence

The cells on the culture plate were first fixed using 4% paraformaldehyde, followed by permeabilization with 0.1% Triton X-100 for 20 min and blocking with 3% bovine serum albumin for 30 min at room temperature. Subsequently, the cells were incubated at 4 °C overnight with an anti-Twist1 antibody (rabbit, 1:200; Proteintech, Wuhan, China). Afterward, the cells were stained using a three-color multiplex fluorescent staining kit (TYR-570 Plus; Recordbio Biological Technology, Shanghai, China) at room temperature for 10–15 min. After sufficient antibody washing, Hes6 antibody (rabbit, 1:200; immunoway; Suzhou, China) was added dropwise to the cells, followed by overnight incubation, and then secondary staining (TYR-520 Plus) was performed. DAPI was used to stain the nuclei, and imaging was performed using a confocal laser scanning microscope (Leica SP8, Germany) with a 63 × objective lens (more detailed parameter information is listed in Table S3). Images were acquired sequentially through three different channels, captured individually, and then merged for analysis and plotted as fluorescence intensity curves.

### RNA sequencing and bioinformatics analysis

HGC 27 cells, stably transfected with Hes6 shRNA and control shRNA (shNC), were collected, and total RNA was subsequently isolated. Transcriptome sequencing was performed using the platform provided by Shanghai Genechem Co., Ltd. (Shanghai, China). Differential expression of the Hes6 gene in both pan-cancer and GC paired samples was analyzed using the GSCA (https://guolab.wchscu.cn//GSCA/)^16^ and TNMplot (https://tnmplot.com/) databases.^17^ Additionally, potential protein interactions with Hes6 were predicted through the BioGRID (https://thebiogrid.org/)^18^ and IntAct (https://www.ebi.ac.uk/intact) databases.^19^

## Statistics and reproducibility

Statistical analyses were performed using GraphPad Prism v9.0.0. All experimental data were obtained from at least three independent, original, and repeated experiments. The results were presented as mean ± standard deviation. Statistical analyses were performed using two-tailed unpaired student's *t*-test, one-way analysis of variance (ANOVA), or two-way ANOVA followed by Tukey's post hoc test. A *P*-value < 0.05 was considered statistically significant and is indicated by asterisk(s) (∗) in the figures.

## Results

### Hes6 expression is elevated in GC tumor tissues and is associated with patient prognosis

**Figure 1:**
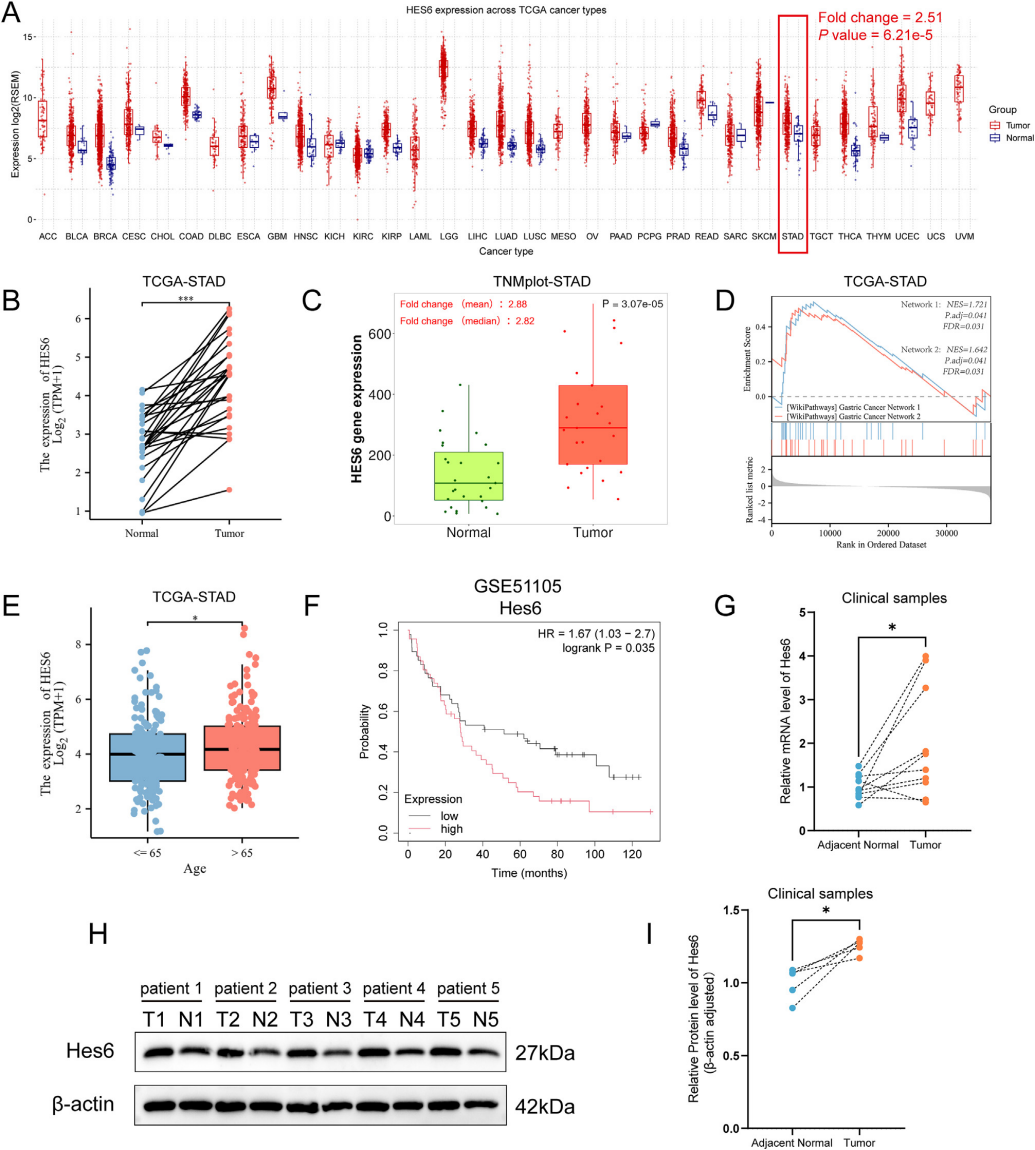
The expression of Hes6 is frequently up-regulated in tumor tissues of human gastric cancer (GC), and is associated with prognosis. **(A)** The pan-cancer expression profile of Hes6 in the TCGA data from the GSCA database showed that Hes6 was up-regulated in GC. **(B)** Hes6 expression was up-regulated in paired GC data from TCGA. **(C)** The paired data expression map of the TNMplot database also confirmed the high expression of Hes6 in GC. **(D)** Gene set enrichment analysis (GSEA) of GC-related marker genes based on HES6 expression in GC data from the TCGA database. **(E)** Difference of HES6 expression in GC of different age groups based on the TCGA database. **(F)** Hes6-related overall survival curve of the GSE51105 dataset. **(G)** Real-time quantitative PCR (RT-qPCR) results of clinical tissue samples from 10 GC patients. **(H)** Western blotting results of clinical tissue samples from 5 GC patients. **(I)** Paired comparison statistics of Western blotting results of clinical tissue samples. ns, not significant; ∗*p* < 0.05, ∗∗*p* < 0.01, and ∗∗∗*p* < 0.001.

To explore the role of Hes6 in gastric carcinogenesis, we initially utilized public databases, especially GSCA and TNMplot, as well as bioinformatics methods to conduct some analyses. Our analysis of Hes6 expression across various cancers using the TCGA database revealed its up-regulation in multiple solid tumors, including bladder, breast, colon, liver, lung, and GC (Fig. 1A). Paired analysis of GC datasets from the TCGA and TNMplot databases revealed that the expression of Hes6 was notably higher in GC tissues compared with normal tissues (Fig. 1B, C). Gene set enrichment analysis (GSEA) further indicated that marker genes associated with GC were significantly enriched in the Hes6 high-expression group (Fig. 1D). Moreover, additional analysis of the correlation between Hes6 expression and the clinical pathological features of GC showed that elevated Hes6 expression was linked to age (Fig. 1E), but not with histological grade (Fig. S1A). Survival analysis demonstrated that in some GC cohorts, higher Hes6 expression was found to correlate with reduced overall survival (Fig. 1F). To further investigate the diagnostic potential of Hes6 as a molecular marker in GC, a receiver operating characteristic curve was generated. The results showed that the value for the area under the curve was 0.825, indicating that it has a certain accuracy in predicting the outcome (Fig. S1B). To further validate this discrepancy, we collected a number of tumor samples from patients diagnosed with GC who had undergone surgery. The results, as shown in Figure 1G–I, both RT-qPCR and Western blotting analyses confirmed a significant up-regulation of Hes6 in GC tissues.

In conclusion, the preliminary investigation into Hes6 reveals that its expression is significantly elevated in GC tissues and correlates with poor prognosis. This suggests that Hes6 may play a crucial role in the tumorigenesis and progression of GC, making it a potential molecular target for further research.

### Hes6 enhances the malignant phenotypic characteristics of GC cells *in vitro*

**Figure 2:**
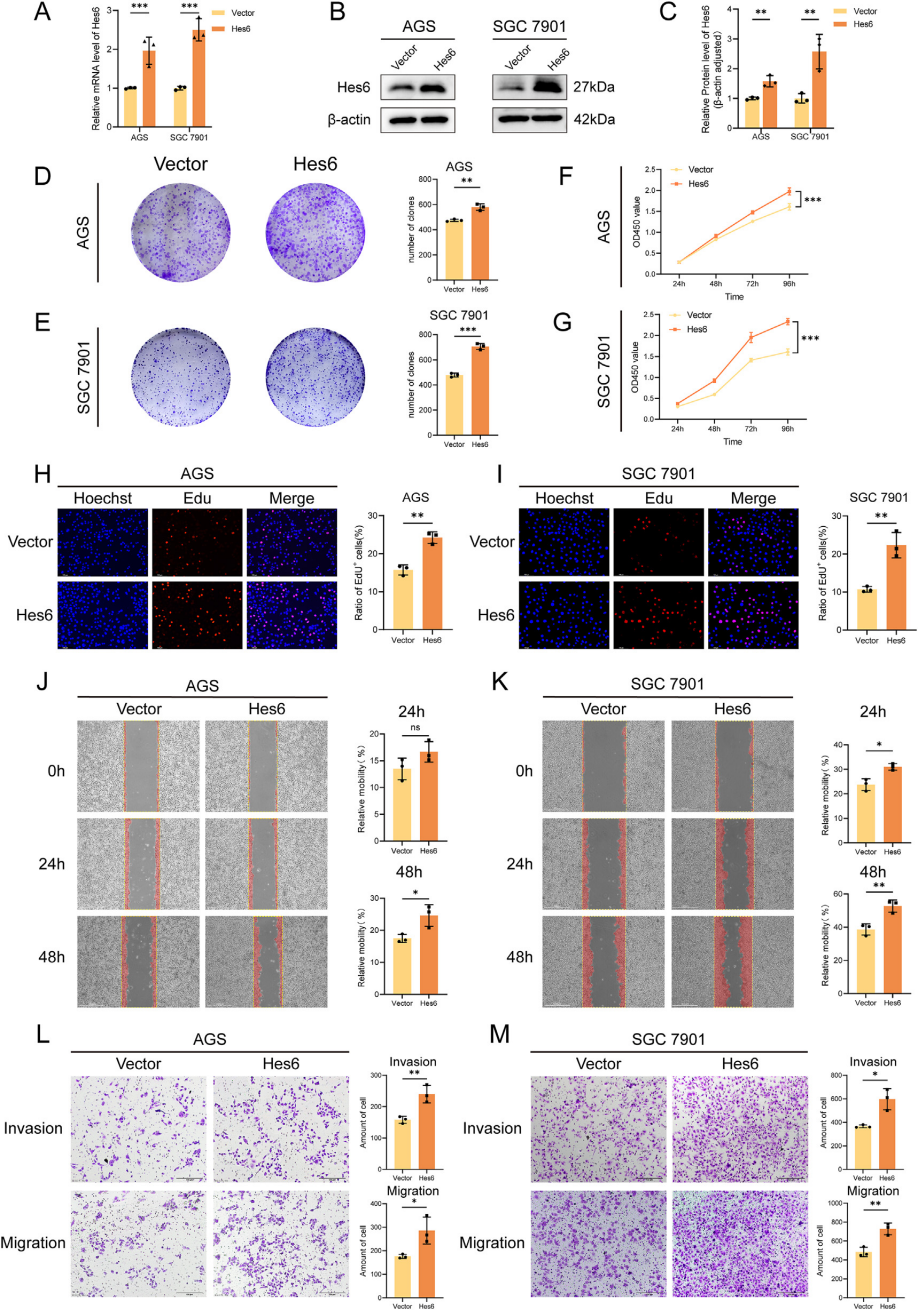
HES6 overexpression promotes proliferation, migration, and invasion phenotypes in GC cells. **(A)** The RT-qPCR results of the Hes6 mRNA levels in GC cell lines AGS and SGC 7901 after infection with lentivirus. **(B, C)** Western blotting results of the Hes6 protein expression levels in GC cell lines AGS and SGC 7901 after transfection, along with the corresponding quantitative statistical results. **(D**–**G)** Representative graphs and quantification of clonogenicity (D, E) and CCK-8 proliferation assays in AGS and SGC 7901 cells stably transfected with vector or Hes6. **(H, I)** The proliferation capacity of AGS and SGC 7901 cells stably transfected with vector or Hes6 was evaluated by EdU assay. **(J**–**M)** Representative graphs and statistical plots of wound assays, as well as transwell assays used to demonstrate the cell migration and invasion ability of AGS and SGC 7901 cells stably transfected with vector or Hes6. ns, not significant; ∗*p* < 0.05, ∗∗*p* < 0.01, and ∗∗∗*p* < 0.001.

**Figure 3:**
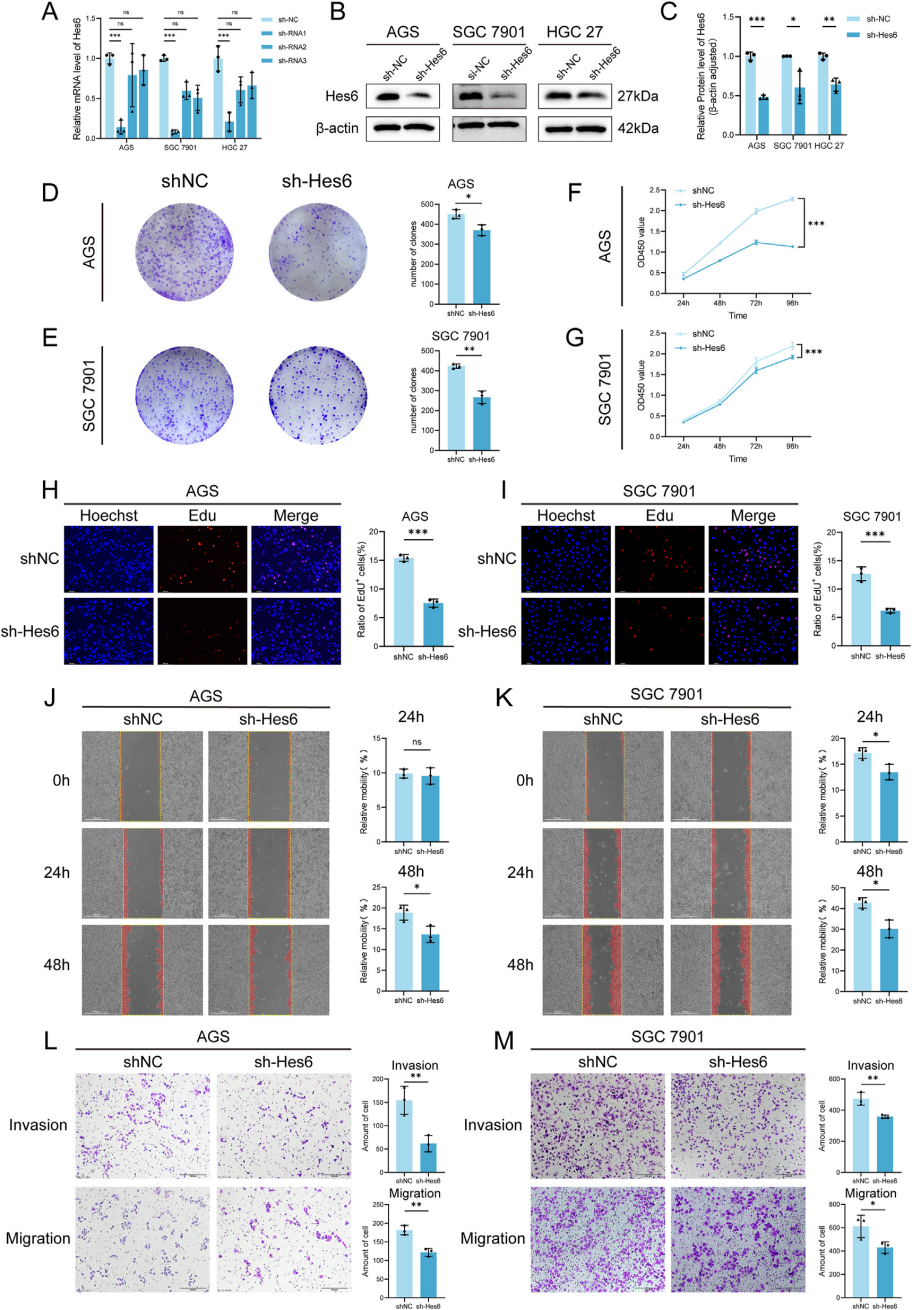
HES6 knockdown inhibits proliferation, migration, and invasion phenotypes in GC cells. **(A)** The RT-qPCR results of the Hes6 mRNA levels in GC cell lines AGS, SGC 7901, and HGC 27 after infection with lentivirus. **(B, C)** Western blotting results of the Hes6 protein expression levels in GC cell lines AGS, SGC 7901, and HGC 27 after transfection, along with the corresponding quantitative statistical results. **(D**–**G)** Representative graphs and quantification of clonogenicity (D, E) and CCK-8 proliferation assays in AGS and SGC 7901 cells stably transfected with shNC or sh-Hes6. **(H, I)** The proliferation capacity of AGS and SGC 7901 cells stably transfected with shNC or sh-Hes6 was evaluated by EdU assay. **(J**–**M)** Representative graphs and statistical plots of wound assays, as well as the transwell assay used to demonstrate the cell migration and invasion ability of AGS and SGC 7901 cells stably transfected with shNC or sh-Hes6. ns, not significant; ∗*p* < 0.05, ∗∗*p* < 0.01, and ∗∗∗*p* < 0.001.

At the same time, to further elucidate the function of Hes6 in the onset and progression of GC, the plasmids designed for the stable overexpression of Hes6 were introduced into the GC cell lines AGS and SGC 7901, while the plasmids aimed at stably knocking down Hes6 were introduced into the AGS, SGC 7901, and HGC 27 cell lines through lentiviral infection experiments. The successful overexpression and knockdown of Hes6 were validated using RT-qPCR and Western blotting analysis (Fig. 2A–C, 3A–C); thereby, stable overexpression and knockdown cell lines were successfully established. Subsequently, we conducted a series of experiments to evaluate the effects of Hes6 overexpression on the proliferation, migration, and invasion capabilities of AGS and SGC 7901 cells, as well as the effects of Hes6 knockdown on the same capabilities in AGS, SGC 7901, and HGC 27 cells.

The plate cloning experiment revealed that Hes6 promoted the capacity of cells to generate monoclonal colonies (Fig. 2D, E). The CCK-8 proliferation assay results also confirmed that cells in the overexpression group exhibited a markedly enhanced proliferation rate (Fig. 2F, G). Additionally, the EdU experiment provided evidence that a higher percentage of positive proliferating cells emerged after Hes6 was overexpressed (Fig. 2H, I). In contrast, in Figure 3D–I and Figure S2A–S2C, the cells that stably knock down Hes6 after lentiviral infection had opposite alterations in the same cell function experiments as before. Meanwhile, gene association analysis demonstrated that Hes6 was significantly associated with tumor proliferation-related signatures (Fig. S1D). These indicate that Hes6 participates in the malignant development of GC cells by enhancing cell proliferation.

Another significant characteristic of cancer cells is their propensity for hematogenous metastasis. Consequently, we proceeded to conduct wound healing and transwell assays to assess the impact of Hes6 overexpression and knockdown on the invasive and migratory capabilities of GC cells. As illustrated in Figure 2J, K, the cells subjected to the overexpression group exhibited a increased migration rate in comparison to the control group. Additionally, the transwell assay demonstrated that a greater number of cells migrated or invaded into the lower chamber following the stable overexpression of Hes6 in the AGS and SGC 7901 cell lines (Fig. 2L, M). Conversely, a reduction in migration and invasion was observed in the presence of Hes6 knockdown, which aligns with the findings from the proliferation assays (Fig. 3J–M; Fig. S2D and S2E). These findings indicate that Hes6 may contribute to the augmentation of the malignant invasive properties of GC cells.

To evaluate the therapeutic potential of targeting Hes6, we employed RNA interference to suppress Hes6 expression and subsequently examined apoptosis in AGS and SGC 7901 cells using annexin V/PI staining. The results demonstrated a significant increase in apoptotic rate upon Hes6 knockdown (Fig. S3D), further substantiating the oncogenic role of Hes6 in GC progression.

### Hes6 binds to Twist1 and maintains the stability of Twist1, promoting EMT of GC cells

**Figure 4:**
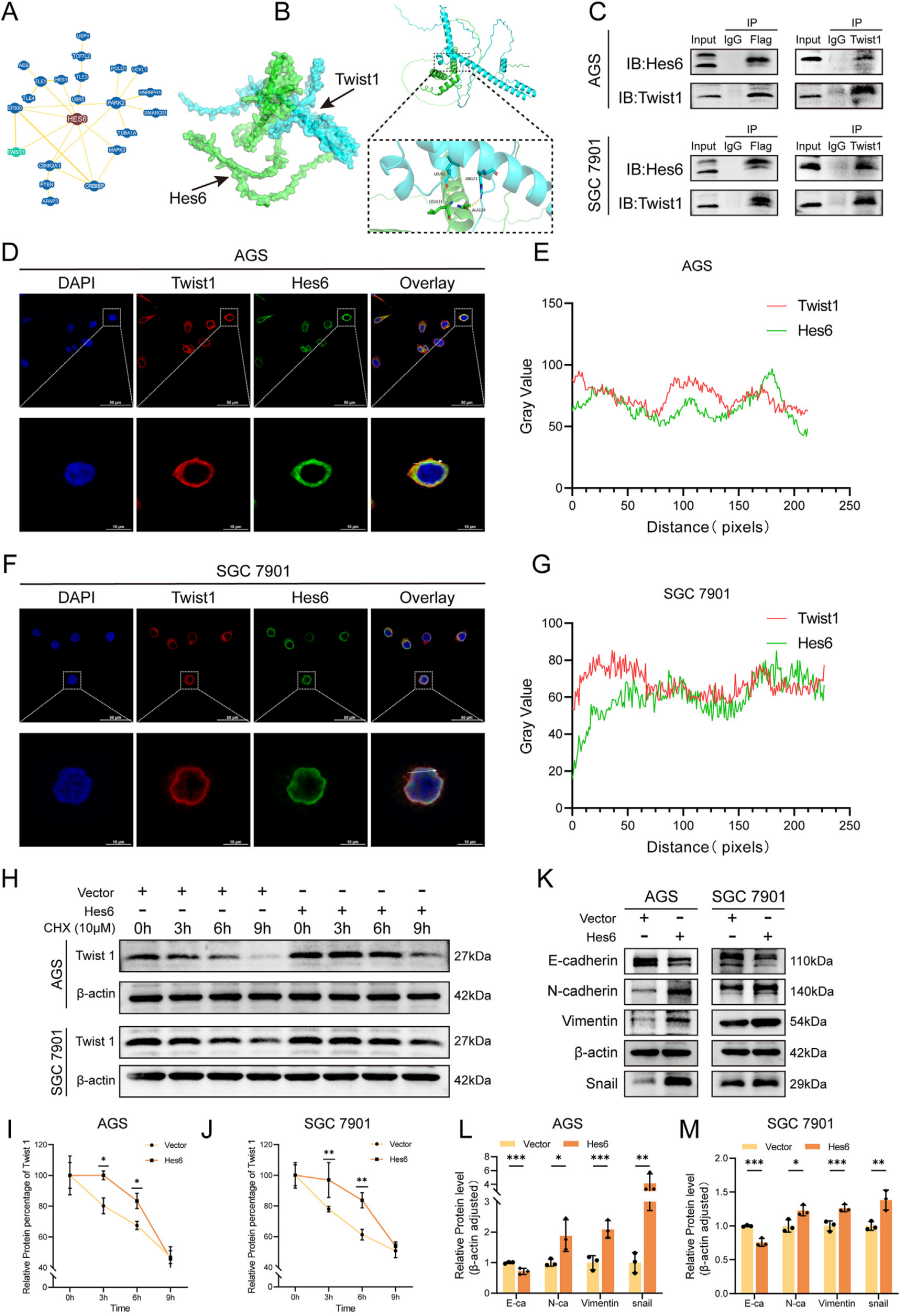
Hes6 binds to Twist1 and maintains the stability of Twist1, promoting epithelial-mesenchymal transition (EMT) of GC cells. **(A)** The network diagram of potential Hes6 binding proteins from the BioGRID database. **(B)** The Hes6-Twist1 protein interaction pattern constructed by computer simulation and molecular docking, and the prediction of potential sites on the interface between the two molecules. **(C)** The interaction between Hes6 and Twist1 in AGS and SGC 7901 cells was investigated using an exogenous immunoprecipitation assay (exogenous Hes6 vector carries a Flag tag). **(D**–**G)** The confocal images showing colocalization of Hes6 (green) and Twist1 (red) in AGS and SGC 7901. Nuclei were counterstained with DAPI (blue). The protein fluorescence intensity quantification curve is shown on the right. **(H)** Western blotting assay of Hes6-related Twist1 protein stability. The protein translation blocker cycloheximide (CHX) (10 μM) was used to treat vector control and Hes6 overexpression groups of AGS and SGC 7901 cells, and the rate of Twist1 protein degradation was detected by Western blotting. **(I, J)** Relative degradation curves of Twist1 protein in different treatment groups. **(K)** The expression levels of EMT-related marker proteins in AGS and SGC7901 cells stably transfected with vector and Hes6 were detected by Western blotting. **(L, M)** Relative quantitative statistics of EMT-related marker protein bands. ∗*p* < 0.05, ∗∗*p* < 0.01, and ∗∗∗*p* < 0.001.

Given that Hes6 is identified as a nuclear transcription cofactor, which suggests that such molecules typically collaborate with other transcription factors to enhance the transcriptional activity of target proteins downstream.^20^^,^^21^ To enhance the comprehension of the biological mechanisms by which Hes6 contributes to the progression of GC, a bioinformatics methodology utilizing publicly available protein interaction databases was implemented to forecast the downstream target proteins that interact with Hes6. The predictive outcomes derived from the BioGRID and IntAct databases indicate that Twist1 is a potential interaction protein with Hes6 (Fig. 4A; Fig. S1J). Subsequently, we used the protein modeling prediction function of AlphaFold3 (https://deepmind.google/technologies/alphafold/) to construct an online protein interaction model of Hes6 and Twist1, and analyzed the contact residues and hydrogen bond interactions on the binding surface of the two molecules through molecular docking fitting (Fig. 4B; Table S3).^22^ Furthermore, the expression levels and prognostic relevance of Twist1 in GC were evaluated. The findings revealed that Twist1 was also overexpressed in GC and had a significant association with poor prognosis (Fig. S1E–S1I).

To further substantiate our hypothesis, immunoprecipitation experiments in conjunction with immunofluorescence co-localization experiments *in vitro* were conducted to confirm the interaction between the proteins Hes6 and Twist1, as well as their co-localization within the nucleus. The results indicate that following the introduction of the exogenous Flag-tagged Hes6 plasmid into AGS and SGC 7901 cell lines, Twist1 was detected in the protein samples acquired through immunoprecipitation, and *vice versa* (Fig. 4C). Coincidentally, the results from the immunofluorescence experiments further corroborate the observation that the two proteins co-localize within the nuclei of AGS and SGC 7901 cells (Fig. 4D–G). This provides us with substantial evidence to believe that these proteins engage in a direct interaction within the cellular environment.

It is widely recognized that the interaction of two proteins to form a complex typically correlates with increased biological activity.^23^ To investigate whether Hes6 enhanced the transcriptional activity of Twist1 by increasing its protein stability, we employed cycloheximide to block the initiation of protein synthesis. After interfering with Hes6 expression, we detected the levels of Twist1 through Western blotting, as illustrated in Figure 4H–J. It was noted that increased levels of Hes6 resulted in a diminished rate of natural degradation of Twist1 in AGS and SGC 7901 cell lines with stable overexpression of Hes6, suggesting that Hes6 contributes to the stabilization of Twist1.

The oncogenic function of Twist1 and its associated mechanisms have been extensively documented in the literature, with particular emphasis on its role as a crucial regulator of EMT.^24^ Consequently, we proceeded to investigate the impact of Hes6 on EMT-related molecules in AGS and SGC 7901 cell lines that exhibit stable overexpression of Hes6. The results demonstrated that Hes6 overexpression induced consistent alterations in major EMT markers at both mRNA and protein levels, showing decreased E-cadherin expression along with increased levels of N-cadherin, vimentin, and Snail (Fig. 4K–M; Fig. S3B and S3C). This indicates that Hes6 induces a transition in the epithelial-mesenchymal characteristics of the cells, with a higher mesenchymal characteristic that may render tumor cells more prone to distant metastasis. This provides a novel perspective for understanding the regulation of EMT in GC cells by the Hes6-Twist1 complex.

Overall, Hes6 is proposed to act as a transcriptional cofactor for Twist1, potentially enhancing its stability and contributing to the regulation of EMT in GC cells, thereby influencing tumor metastasis.

### Hes6 contributes to GC progression by regulating the PI3K/AKT signaling pathway *in vitro*

**Figure 5:**
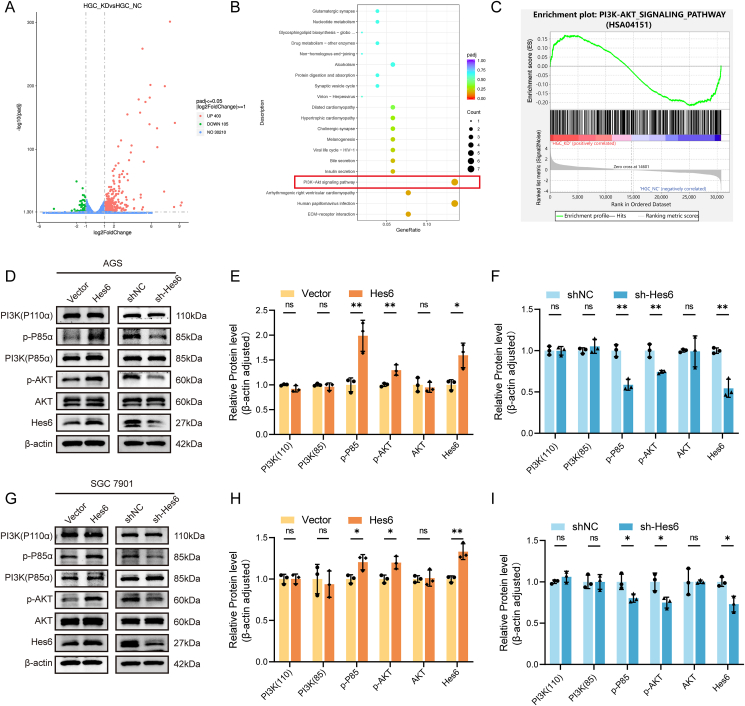
RNA sequencing reveals that Hes6 expression may be associated with the PI3K/AKT signaling pathway. **(A)** RNA sequencing results showed genes with significant expression differences between HGC-KD and HGC-NC groups, presented as a volcano plot. **(B)** KEGG functional enrichment analysis showed potential molecular signaling pathways associated with Hes6 knockdown. **(C)** Gene set enrichment analysis (GSEA) confirmed that the PI3K/AKT signaling pathway was significantly related to Hes6. **(D**–**I)** Western blotting verified the changing trend of PI3K/AKT signaling pathway-related proteins accompanied by the intervention of Hes6 expression in AGS and SGC 7901 cells. The relative quantitative statistics of protein expression among different groups were presented. ns, not significant; ∗*p* < 0.05, ∗∗*p* < 0.01, and ∗∗∗*p* < 0.001.

To investigate the molecular mechanisms underlying the role of Hes6 in GC pathogenesis, we conducted transcriptome sequencing on HGC 27 cells with stable Hes6 knockdown. The sequencing analysis revealed significant changes in the expression of 505 genes, with 105 genes showing notable down-regulation in the knockdown group compared with the control group. (|Log_2_ fold change | ≥ 1, *P*_adj_ < 0.05) (Fig. 5A). The subsequent Kyoto Encyclopedia of Genes and Genomes (KEGG) enrichment analysis revealed a significant enrichment of the PI3K/AKT signaling pathway, with a higher number of relevant genes identified in the knockdown group (Fig. 5B; *P* < 0.05). Furthermore, GSEA corroborated a positive association between this signaling pathway and Hes6 (Fig. 5C). Therefore, we expect to further explore the impact of intervening in Hes6 expression on molecules related to the PI3K/AKT signaling pathway *in vitro* through Western blotting. Interestingly, the findings indicate that phosphorylated PI3K protein (phosphorylated P85) and phosphorylated AKT protein significantly increased in both AGS and SGC 7901 cells following Hes6 overexpression, whereas the non-phosphorylated proteins PI3K (110α), PI3K (85α), and AKT remained insignificantly altered (Fig. 5D–I). In contrast, the aforementioned proteins showed entirely opposite alterations in AGS and SGC 7901 cells with Hes6 knockdown (Fig. 5D–I). These results suggest that the variations in Hes6 expression align with the PI3K/AKT pathway's activation or inhibition.

### Blocking the PI3K/AKT signaling pathway will suppress the Hes6-mediated pro-GC cell phenotype

**Figure 6:**
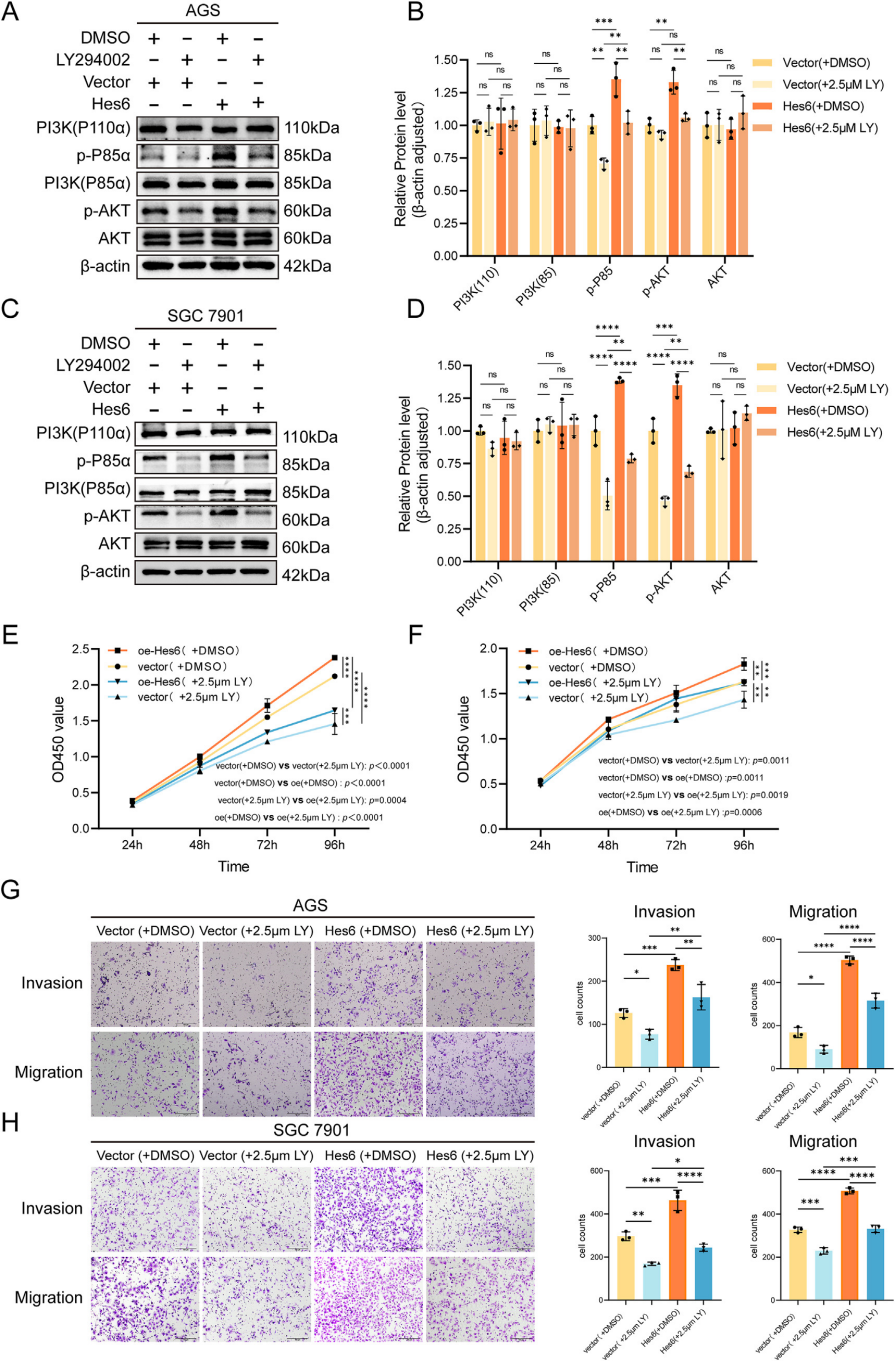
Blockade of PI3K/AKT signaling by the PI3K inhibitor LY294002 impairs Hes6-induced tumorigenesis in GC. **(A**–**D)** The PI3K/AKT signaling pathway protein levels of Hes6-transfected GC cells treated with the LY294002 inhibitor were detected by Western blotting analysis, with relative quantitative statistical plots of protein bands. **(E, F)** CCK-8 proliferation assay was performed on Hes6-transfected GC cells treated with the LY294002 inhibitor. **(G, H)** The invasion and migration abilities of Hes6-transfected GC cells treated with LY294002 inhibitor were assessed by transwell assay, and the number of relative invading/migrating cells was statistically analyzed. ns, not significant; ∗*p* < 0.05, ∗∗*p* < 0.01, ∗∗∗*p* < 0.001, and ∗∗∗∗*p* < 0.0001.

To obtain more robust evidence concerning the regulation of GC cell functions by the Hes6-mediated PI3K/AKT signaling pathway, we employed the broad-spectrum PI3K inhibitor LY294002 to selectively inhibit the activity of PI3K/AKT pathway proteins (primarily their phosphorylated forms) *in vitro*, and verified the protein expression levels of molecules in the PI3K/AKT signaling pathway post-treatment via Western blotting analysis (Fig. 6A–D).

Subsequent cellular function experiments proved that the presence of LY294002 in both the vector group and the overexpressed Hes6 group resulted in a significantly reduced cell proliferation curve in the CCK-8 assay compared with the DMSO group (Fig. 6E, F), while the transwell assay indicated an evident inhibition of cell invasion and migration capabilities (Fig. 6G, H). Although the overexpression group appears to exhibit greater strength than the vector group in cell proliferation, migration, and invasion following LY294002 treatment, it is unequivocally clear that inhibiting the PI3K/AKT signaling pathway effectively attenuates the Hes6-induced enhancement of malignant traits in GC cells.

### Hes6 is associated with tumor formation and growth *in vivo*

**Figure 7:**
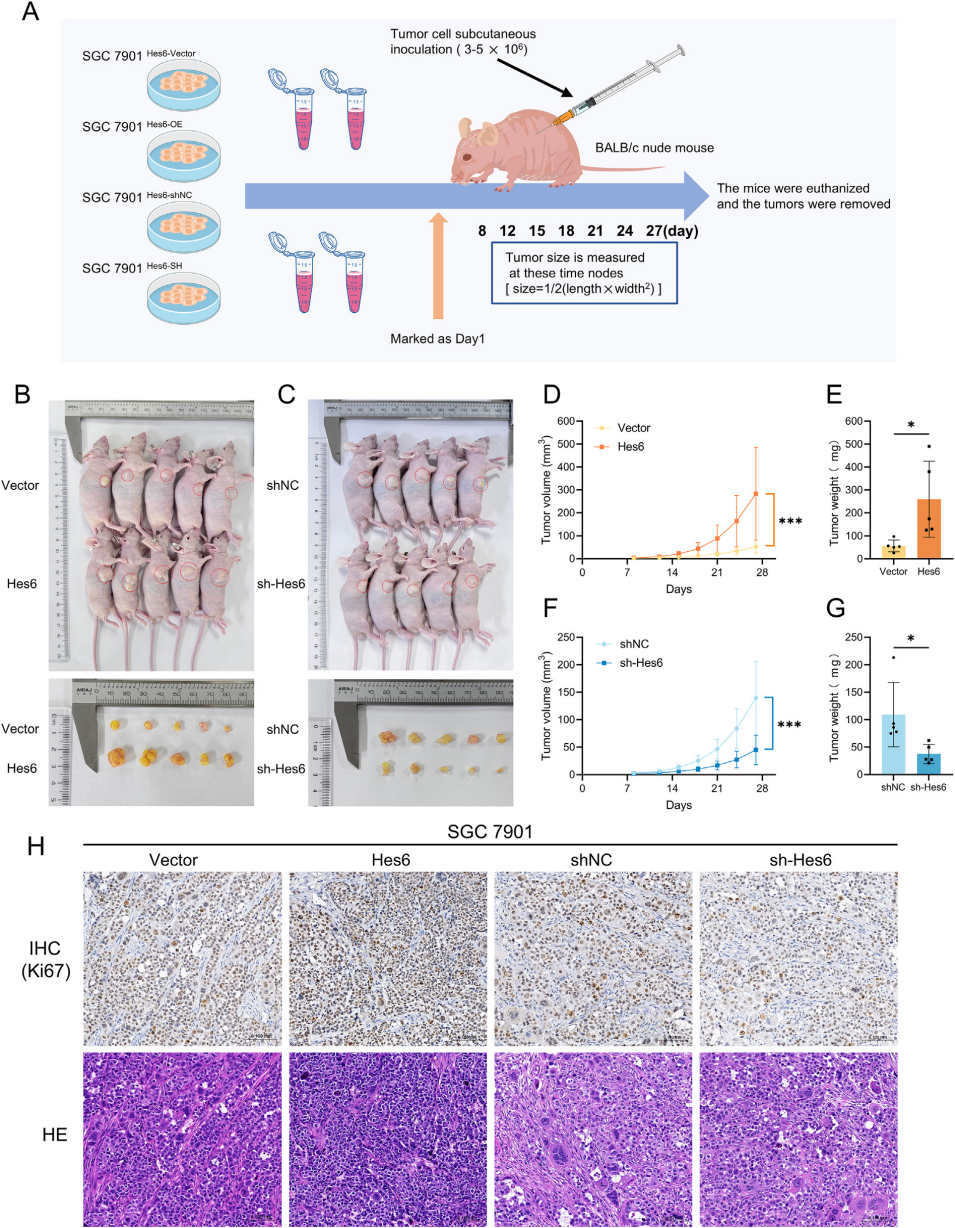
Hes6 promotes GC tumor growth *in vivo*. **(A)** Schematic diagram of subcutaneous tumor implantation in nude mice. **(B, C)** Representative images of xenograft tumors. **(D, E)** The growth curves of xenograft tumors and comparison of tumor weights in mice implanted with SGC 7901^Vector^ and SGC 7901^Hes6^ cells (*n* = 5). **(F, G)** The growth curves of xenograft tumors and comparison of tumor weights in mice implanted with SGC 7901^shNC^ and SGC 7901^sh−Hes6^ cells (*n* = 5). **(H)** Representative images of immunohistochemistry staining with anti-Ki67, as well as related HE-stained images in xenograft tumors of different groups. ∗*p* < 0.05, ∗∗*p* < 0.01, and ∗∗∗*p* < 0.001.

To examine the influence of Hes6 on tumorigenesis *in vivo*, we performed animal studies utilizing a cell-derived xenograft model in nude mice. As depicted in the schematic diagram (Fig. 7A), after inoculating the stable transfected SGC 7901 cells overexpressing/knocking down Hes6 and the corresponding control group cells into the subcutaneous tissue of mice, the group with Hes6 overexpression demonstrated the highest rates of tumor growth, as well as the largest average tumor volume and weight among the four experimental groups (Fig. 7B–D, E). Conversely, the group with Hes6 knockdown displayed the slowest growth rate, along with the smallest average tumor volume and weight (Fig. 7C–F, G). Furthermore, the immunohistochemistry experiment is employed to evaluate the expression levels of Ki-67 in subcutaneous tumors across various groups, thereby facilitating a more comprehensive assessment of the proliferative potential of tumor cells *in vivo*. As shown in Figure 7H, the results reveal that Ki-67 expression is maximized in the overexpression group and minimized in the knockdown group, which is a favorable support for the previous results. Collectively, these findings provide further evidence for the assertion that Hes6 facilitates the proliferation of GC cells *in vivo*.

## Discussion

GC is one of the most common gastrointestinal neoplasms, highlighted by advanced invasiveness and poor prognosis, arising and progressing due to the interplay of multiple complex variables, including but not limited to environmental, physicochemical, and genetic influences.^2^^,^^3^^,^^25^ Genetic changes, a prevalent phenomenon associated with cancer development, serve as significant driving factors for tumor heterogeneity, playing an essential role in carcinogenesis.^26^ It is generally recognized that transcription is a key step in gene expression, which is regulated by numerous transcription factors, cofactors, and chromatin-modifying proteins. The dysregulation of specific gene expression may occur and develop from an initial imbalance in transcriptional regulation, ultimately leading to the occurrence of cancer and various diseases.^27^ Among them, due to the lack of specific domains, transcription cofactors cannot bind to DNA directly but instead exert their function of activation or inhibition by binding to or interacting with transcription factors through “auxiliary” characteristics, which makes them seem to have attracted little attention.^28^, ^29^, ^30^ Nevertheless, recent research has introduced new perspectives by lifting the veil of novel and crucial functions about cofactors in DNA loop creation and maintenance, which is essential for delicate gene regulation.^31^ Classic transcription cofactors are divided into several major families, such as BET, SRC, KMT, CBP/p300, CRTC, CITED, TRIM, and MRTF family.^28^^,^^32^ For example, YAP is one of the most famous transcription activators and a proto-oncogene. It is widely recognized that YAP functions as a transcriptional co-activator and a downstream effector of the Hippo signaling pathway. It regulates gene expression through binding to TEAD family transcription factors and is thought to play a significant role in tumor initiation and progression.^33^, ^34^, ^35^

In summary, the mechanism refers to the special function of cofactors in gene expression control, which may represent an important oncogenic driver in gastric carcinogenesis, a component that has often been overlooked in the past. Investigating the function of particular genes in tumors and comprehending the interactions between cofactors and transcription factors, as well as how these intricate apparatuses govern gene regulation and functional alterations in cancer, will be crucial for deepening our understanding of tumor characteristics and aiding in the development of more precise and targeted treatment approaches.

In this study, we have discovered that Hes6 functions as a transcriptional cofactor that is up-regulated in GC, promoting enhanced proliferation, migration, and invasion in GC cells. Actually, Hes6 was first recognized for its positive role in neuronal differentiation.^36^ Mechanistically, Hes6 promotes neuronal differentiation through enhancing the biochemical activity of proneuronal proteins, and the interaction between Hes6 and drosophila groucho (Gro)/transducin-like enhancer of split (TLE) enables the inhibition of astrocyte differentiation.^37^ Subsequently, several studies clarified the role of Hes6 in different cancers. A study performed by Pandiani et al sought to tackle the critical issue of primary uveal melanoma cell heterogeneity through single-cell transcriptome analysis and revealed that Hes6 could enhance the invasive capacity and motility of uveal melanoma, consequently facilitating the metastatic dissemination of primary uveal melanoma cells.^38^ Ramos-Montoya A's research has demonstrated that Hes6, a crucial transcriptional cofactor, not only synergistically boosts E2F1 activity in castrate-resistant prostate cancer but is also vital for sustaining androgen receptor function, enabling cellular proliferation in the absence of testosterone.^39^ Consistent with this research, our preliminary exploration broadens the understanding of Hes6's role in cancer. Likewise, earlier research has highlighted the activation of various transcriptional cofactors in GC, shedding light on their mechanisms of action. For instance, disruption of the Hippo signaling pathway in gastrointestinal tissues is often a key factor in the development of *H. pylori*-induced GC. Following *H. pylori* infection, there is an increase in the nuclear expression of TAZ and enhanced transcriptional activity of the TEAD transcription factor, which in turn promotes EMT, invasion, and stemness in cancer cells.^40^^,^^41^

We further confirmed that Twist1 was a direct target of Hes6 through database screening combined with experimental validation *in vitro*, and Hes6 could bind to Twist1 as a transcriptional cofactor to enhance its stability. Moreover, we investigated the impact of Hes6 on the EMT process, particularly considering the well-established role of Twist1 in regulating EMT. EMT has been widely recognized as a critical driver of primary tumor metastasis, promoting cancer progression through various mechanisms, including enhancing tumor cell motility, disrupting apical-basal polarity, and fostering drug resistance.^42^^,^^43^ Furthermore, numerous studies have highlighted the regulatory roles and mechanisms of diverse intercellular interactions within the tumor microenvironment, as well as transcriptional elements, post-translational modifications, and cellular signaling pathways in the EMT network.^44^^,^^45^ Twist1, initially identified as a key zygotic gene in early embryonic development of drosophila, encodes a transcription factor containing a basic helix-loop-helix (bHLH) domain.^46^^,^^47^ Genetic studies have fully emphasized the pivotal role of Twist1 in mesodermal development.^48^ Another striking function refers to its significant role in promoting cancer metastasis. Specifically, Twist1 represses E-cadherin expression through the recruitment of nucleosome remodeling and deacetylase complexes, leading to gene silencing. Additionally, Twist1 enhances the expression of factors such as Bmi1, AKT2, and YB-1, which collectively contribute to the promotion of EMT and finally induce the enhanced ability of cancer cell migration, invasion, stemness, and drug resistance.^49^^,^^50^ Coincidentally, it has been reported that other molecules enhance Twist1 stability to promote EMT and endow cancer cells with strengthened metastasis potential, which concretizes our conclusion as well.^51^

According to the integrated results from RNA sequencing, we assessed and proposed that the PI3K/AKT signaling pathway played a critical role in mediating the effects of Hes6 on the malignant characteristics of GC cells. Subsequent experimental analysis *in vitro* further confirmed that the activation/inhibition state of the PI3K/AKT signaling pathway was affected by Hes6 expression and that specific blocking of these signaling pathway molecules reversed the proliferation and invasion ability of cancer cells conferred by Hes6. It is well-established that the PI3K/AKT signaling pathway is a highly conserved regulatory network that governs key cellular processes such as growth, proliferation, and differentiation in eukaryotic cells.^52^^,^^53^ The dysregulation of this pathway usually drives the occurrence and development of malignancies. It participates in tumorigenesis, proliferation, apoptosis, EMT, oscillation of the immune microenvironment, and drug resistance. While approximately half of tumors develop with aberrant PI3K/AKT signaling pathways,^53^^,^^54^ previous studies have reported the mechanism by which transcription cofactors participate in cancer progression through the PI3K/AKT signaling pathway. For example, CRTC2 is a protein that is highly expressed in tumor tissues of individuals with chemotherapy-resistant ovarian cancer, and it can partially control autophagy flux through the PI3K/AKT pathway to affect tumor progression.^55^ To our knowledge, this is the first evidence indicating that transcription cofactors contribute to the progression of GC through the PI3K/AKT signaling pathway.

**Figure 8:**
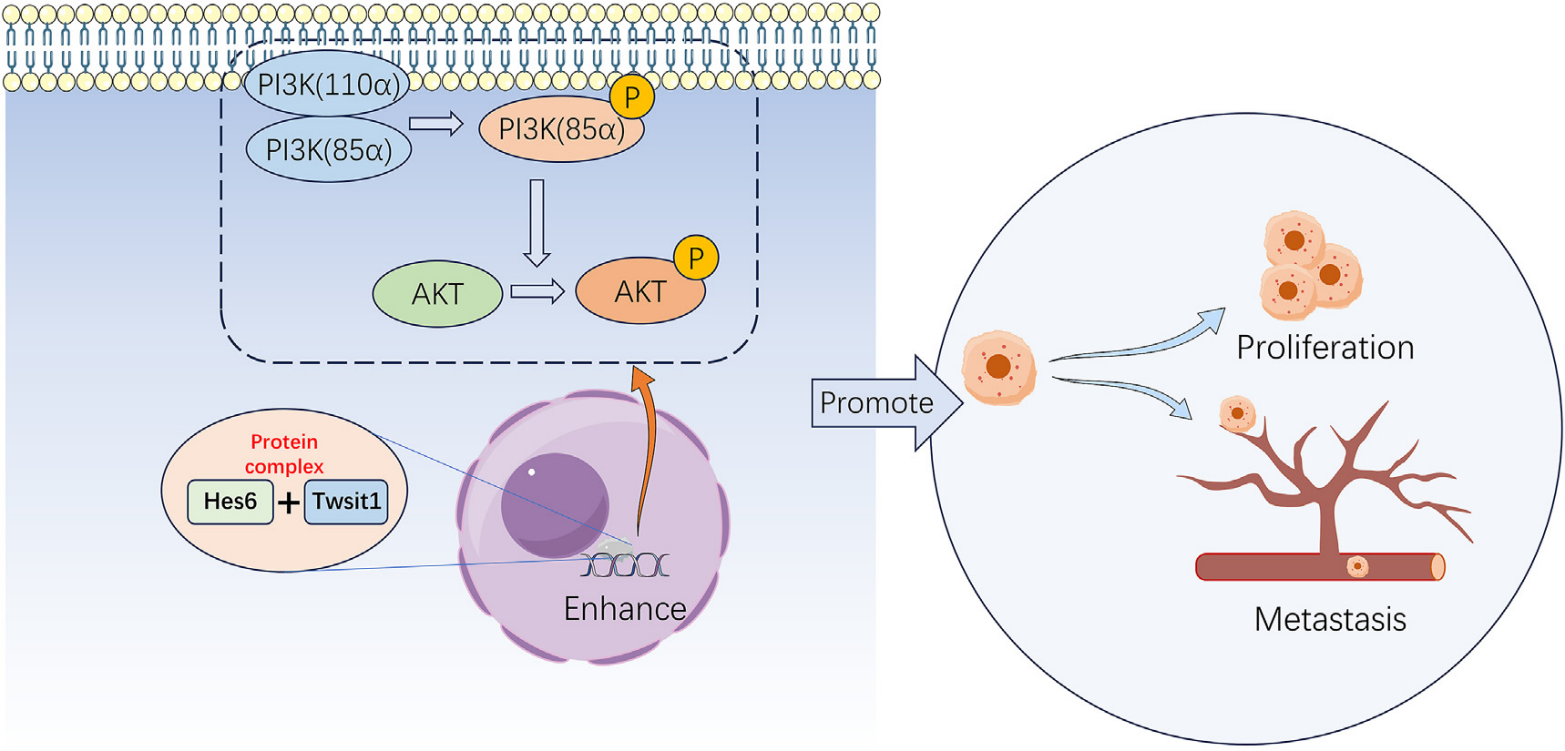
Schematic illustration of the potential mechanism by which abnormally expressed Hes6 promotes cell proliferation and tumorigenesis of GC.

In summary, on the one hand, our results reveal the stabilizing positive effect of Hes6 on Twist1 and its impact on EMT, and on the other hand, also highlight the unique role and potential mechanism of Hes6 in regulating GC cell proliferation and invasion (Fig. 8). This study provides a new perspective on the pathogenesis of GC and offers valuable scientific insights into potential therapeutic strategies targeting this disease. However, limitations remain. The content of the current study is not sufficient, with obvious deficiencies in research logic, depth of molecular mechanisms, and the persuasiveness of the model. In the future, it is necessary to verify it through additional *in vivo* models, explore the possible off-target effects of Hes6 inhibition, and analyze other related signaling transduction mechanisms to find and establish a theoretical foundation for the expectation on Hes6 as a therapeutic target for GC.

## CRediT authorship contribution statement

**Can Fang:** Writing – original draft, Project administration, Methodology, Investigation, Formal analysis, Data curation, Conceptualization. **Zhiwei Peng:** Writing – original draft, Investigation, Formal analysis, Data curation, Conceptualization. **Yaru Sang:** Software, Formal analysis, Data curation. **Zihao Ren:** Software, Formal analysis, Data curation. **Kang Wang:** Software, Formal analysis, Data curation. **Nuo Xu:** Software, Formal analysis, Data curation. **Ying Li:** Software, Formal analysis, Data curation. **Tingting Guo:** Software, Formal analysis, Data curation. **Yinan Zhu:** Software, Formal analysis, Data curation. **Shangxue Yan:** Writing – review & editing, Supervision, Resources, Methodology, Data curation, Conceptualization. **Kongwang Hu:** Writing – review & editing, Supervision, Project administration, Methodology, Investigation, Funding acquisition, Conceptualization.

## Ethical statement

GC tissues and corresponding adjacent normal tissues used in the experiments were collected from surgical specimens of GC patients who provided informed consent at the First Affiliated Hospital of Anhui Medical University. This study was conducted in compliance with the Declaration of Helsinki and received approval from the Ethics Committees of the First Affiliated Hospital of Anhui Medical University (2023658). All animal procedures were approved by the Experimental Animal Ethics Committee of Anhui Medical University (No. LLSC20241788).

## Data availability

The datasets produced in this study can be obtained from the corresponding author upon reasonable request.

## Funding

This study was funded by the Key Scientific Research Projects of Anhui Higher Education Institutions in 2023 (China) (No. 2023AH050573), the 2023 Anhui Province Fuyang City Health Research Project (China) (No. FY2023-027), the Key Research and Development Project of the Anhui Provincial Department of Science and Technology (China) (No. 202004j07020036), and the Key Teaching Research Project of the Anhui Provincial Department of Education (China) (No. 2020xsxxkc247).

## Conflict of interests

The authors declare no known financial or personal conflict of interests that could have influenced the work presented in this paper.

## Figures and Tables

**Figure:**
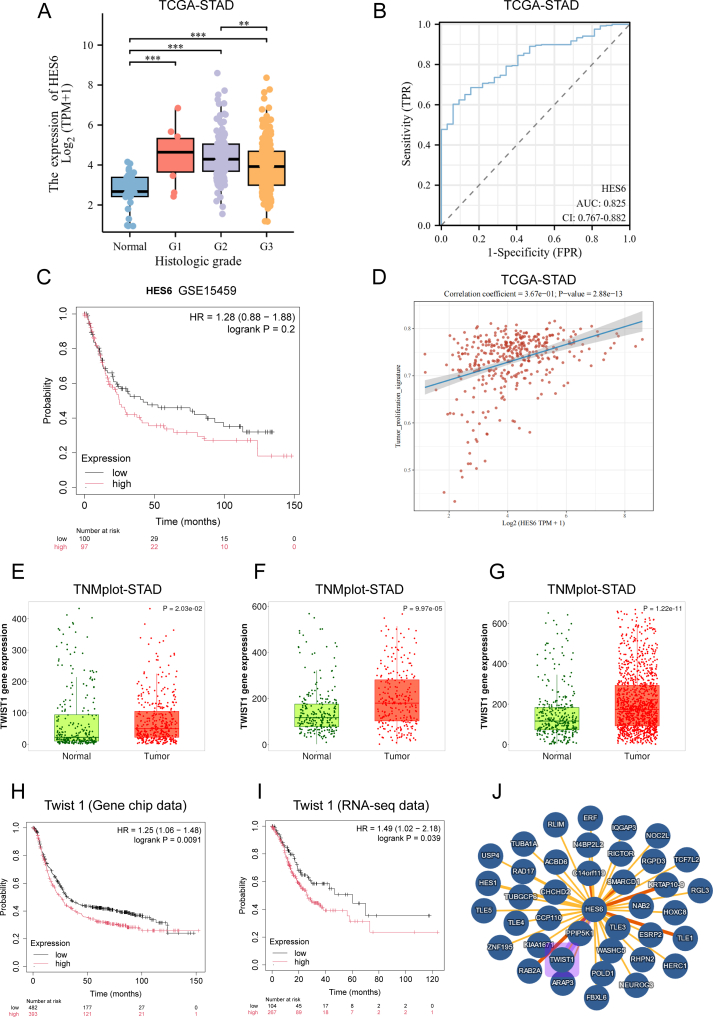
**Figure S1** Clinical pathological bioinformatics analysis of Hes6 and Twist1 in gastric cancer (GC). **(A)** Expression of Hes6 in GC of different histologic grades. **(B)** The receiver operating characteristic curve of Hes6 in GC. **(C)** Hes6-related survival curves of the GSE15459 dataset. **(D)** Scatter plot of correlation between Hes6 and tumor proliferation signatures. **(E)** Expression of Twist1 in GC and normal tissues in the TNMplot database (RNA sequencing data). **(F)** Expression of Twist1 in GC and normal tissues in the TNMplot database (paired Gene chip data). **(G)** Expression of Twist1 in GC and normal tissues in the TNMplot database (unpaired gene chip data). **(H)** Twsit1-related survival curves of GC in the KM-plotter database (Gene chip data). **(I)** Twsit1-related survival curves of GC in the KM-plotter database (RNA sequencing data). **(J)** Hes6-associated protein network predicted in the IntAct database. ∗*P* < 0.05, ∗∗*P* < 0.01, and ∗∗∗*P* < 0.001.

**Figure:**
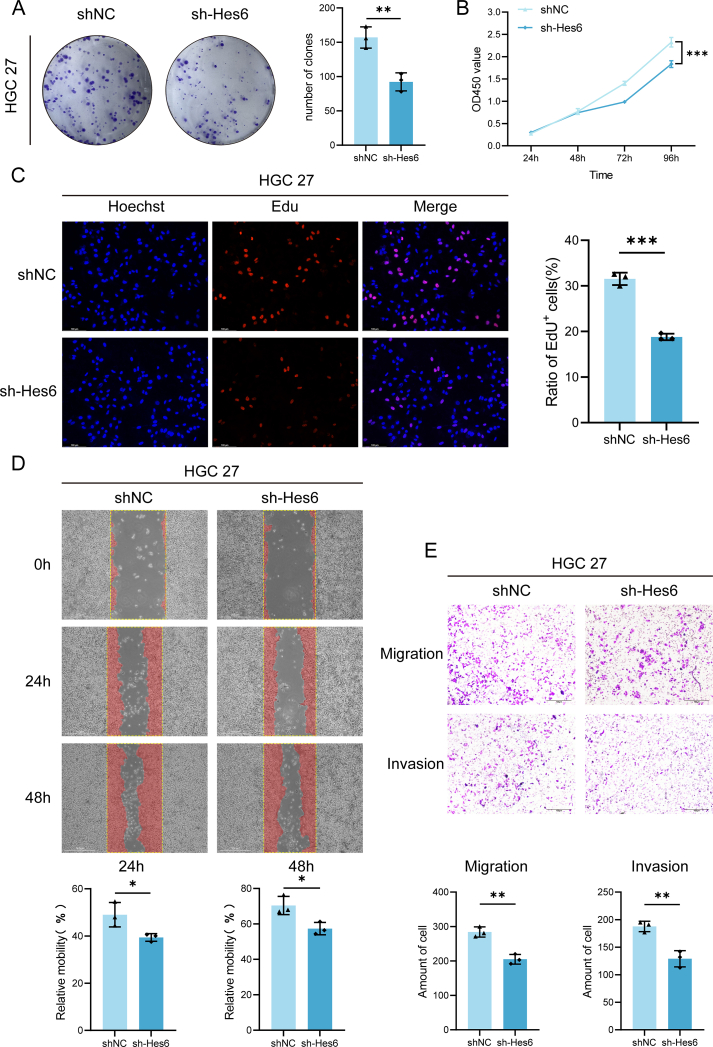
**Figure S2** HES6 knockdown inhibits proliferation, migration, and invasion phenotypes in HGC 27 cells. **(A, B)** Representative graphs and quantification of clonogenicity and CCK-8 proliferation assays in HGC 27 cells stably transfected with shNC or sh-Hes6. **(C)** The proliferation capacity of HGC 27 cells stably transfected with shNC or sh-Hes6 was evaluated by EdU assay. **(D, E)** Representative graphs and statistical plots of scratch assays as well as transwell assays demonstrate the cell migration and invasion ability of HGC 27 cells stably transfected with shNC or sh-Hes6. ∗*P* < 0.05, ∗∗*P* < 0.01, and ∗∗∗*P* < 0.001.

**Figure:**
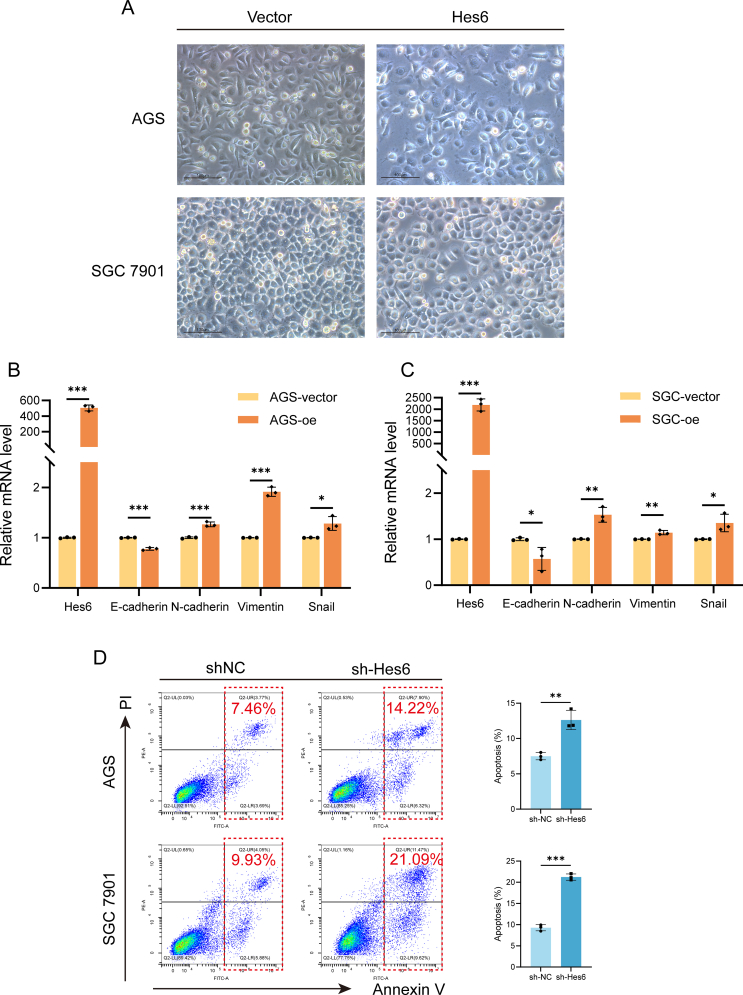
**Figure S3** The effect of Hes6 overexpression on cell morphology and epithelial-mesenchymal transition marker mRNA and the effect of Hes6 inhibition on cell apoptosis. **(A)** Effects of Hes6 overexpression on the morphology of AGS and SGC7901 cells. **(B)** Effects of Hes6 overexpression on mRNA levels of epithelial-mesenchymal transition markers in AGS and SGC7901 cells. **(C)** Effects of Hes6 inhibition on apoptosis of AGS and SGC7901 cells. ∗*P* < 0.05, ∗∗*P* < 0.01, and ∗∗∗*P* < 0.001.
