# Supplementary material for: Transcription cofactor Hes6 interacts with Twist1 to facilitate EMT and promote gastric carcinogenesis by activating the PI3K/AKT signaling
Source: Genes Dis. 2025 May 5;13(5):101674. doi: 10.1016/j.gendis.2025.101674 (PMC13264254; doi:10.1016/j.gendis.2025.101674)
Supplement: Multimedia component 1 [file mmc1.docx]

**Table S1:Clinicopathological data of clinical gastric cancer samples.**

| **Number** | **Sex** | **Age** | **Tumor location** | **Histology grade** | **Size** | **T satge** | **N stage** | **pTNM**  **stage** |
| --- | --- | --- | --- | --- | --- | --- | --- | --- |
| 1 | Male | 60 | Cardia（less curvature） | Adenocarcinoma  （Poorly differentiated） | 2.5cm×2.0cm×0.6cm | T3 | N3a | pT3N3aMx；ⅢB |
| 2 | Male | 65 | Cardia（gastric body） | Adenosquamous carcinoma  （Poorly differentiated） | 5.5cm×5.0cm×3.0cm | T3 | N1 | pT3N1Mx；ⅡB |
| 3 | Female | 55 | Gastric body（less curvature） | Adenocarcinoma  （Poorly differentiated） | 7.0cm×5.0cm×1.0cm | T3 | N3a | pT3N3aMx；ⅢB |
| 4 | Male | 60 | Cardia（less curvature） | Adenocarcinoma  （Moderately to poorly differentiated） | 4.0cm×3.0cm×1.0cm | T3 | N0 | pT3N0Mx；ⅡA |
| 5 | Male | 62 | Gastric antrum （less curvature） | Adenocarcinoma  （Moderately differentiated） | 4.0cm×2.5cm×1.5cm | T3 | N2 | pT3N2Mx；ⅢA |
| 6 | Female | 66 | Cardia | Adenocarcinoma  （Moderately to poorly differentiated） | 5.5cm×4.0cm×1.2cm | T3 | N2 | pT3N2Mx；ⅢA |
| 7 | Male | 65 | Cardia | Adenocarcinoma  （Poorly differentiated） | 4.0cm×2.5cm×1.0cm | T3 | N3a | pT3N3aMx；ⅢB |
| 8 | Male | 69 | Gastric body（less curvature） | Adenocarcinoma  （Poorly differentiated） | 3.0cm×2.5cm×1.0cm | T3 | N0 | pT3N0Mx；ⅡA |
| 9 | Male | 79 | Cardia（less curvature） | Adenocarcinoma  （Poorly differentiated） | 7cm×6.5cm×1.2cm | T4b | N1 | pT4bN1Mx；ⅢB |
| 10 | Male | 45 | Cardia（less curvature） | Adenocarcinoma  （Moderately differentiated） | 3.7cm×2cm×1.0cm | T3 | N0 | pT3N0Mx；ⅡA |
|  | | | | | | | | |
| **Continued table** | | | | | | | | |
| 11 | Male | 73 | Gastric body（less curvature） | Adenocarcinoma  （Poorly differentiated） | 7.5cm×4.0cm×1.7cm | T4a | N3a | pT4aN3aMx；ⅢB |
| 12 | Female | 80 | Cardia（gastric fundus） | Adenocarcinoma  （Moderately differentiated） | 8.0cm×6.0cm×1.8cm | T4b | N1 | pT4bN1Mx；ⅢB |
| 13 | Male | 72 | Cardia（gastric fundus and body） | Mucinous adenocarcinoma  （Poorly differentiated） | 8.0cm×8.0cm×2.0cm | T3 | N2 | pT3N2Mx；ⅢA |
| 14 | Male | 63 | Gastric antrum（less curvature） | Adenocarcinoma  （Poorly differentiated） | 6.0cm×5.0cm×2.0cm | T3 | N3a | pT3N3aMx；ⅢB |
| 15 | Male | 79 | Cardia（less curvature） | Adenocarcinoma  （Poorly differentiated） | 6.0cm×5.0cm×1.0cm | T3 | N2 | pT3N2Mx；ⅢA |
| 16 | Female | 68 | Cardia（less curvature） | Adenocarcinoma  （Moderately to poorly differentiated） | 5.0cm×4.5cm×2.0cm | T3 | N1 | pT3N1Mx；ⅡB |
| 17 | Male | 68 | Cardia（less curvature） | Adenocarcinoma  （Poorly differentiated） | 2.5cm×2.0cm×0.5cm | T3 | N2 | pT3N2Mx；ⅢA |
| 18 | Male | 60 | Cardia（gastric fundus） | Adenocarcinoma  （Moderately differentiated） | 8.0cm×4.0cm×1.0cm | T3 | N0 | pT3N0Mx；ⅡA |
| 19 | Female | 73 | Cardia（less curvature） | Adenocarcinoma  （Poorly differentiated） | 6.0cm×4.0cm×0.8cm | T3 | N3a | pT3N3aMx；ⅢB |
| 20 | Male | 74 | Gastric body（less curvature） | Adenocarcinoma  （Moderately differentiated） | 4.0cm×3.0cm×1.2cm | T3 | N1 | pT3N1Mx；ⅡB |
