# Supplementary material for: Transcription cofactor Hes6 interacts with Twist1 to facilitate EMT and promote gastric carcinogenesis by activating the PI3K/AKT signaling
Source: Genes Dis. 2025 May 5;13(5):101674. doi: 10.1016/j.gendis.2025.101674 (PMC13264254; doi:10.1016/j.gendis.2025.101674)
Supplement: Multimedia component 2 [file mmc2.docx]

**1.Gene cDNA primer sequences used in qPCR experiments (5’ to 3’).**

HES6 (Forward primer): CCC TGA GGC TGA ACT GAG TC

HES6 (Reverse primer): CAA TTT GGG CTG TGG TCA GG

GAPDH (Forward primer): CAG GAG GCA TTG CTG ATG AT

GAPDH (Reverse primer): GAA GGC TGG GGC TCA TTT

β-actin (Forward primer): CAC CAT TGG CAA TGA GCG GTT C

β-actin (Reverse primer): AGG TCT TTG CGG ATG TCC ACG T

E-cadherin (Forward primer):CGA GAG CTA CAC GTT CAC GG

E-cadherin (Reverse primer):GGG TGT CGA GGG AAA AAT AGG

N-cadherin (Forward primer):TTT GAT GGA GGT CTC CTA ACA CC

N-cadherin (Reverse primer):ACG TTT AAC ACG TTG GAA ATG TG

Vimentin (Forward primer):GAG AAC TTT GCC GTT GAA GC

Vimentin (Reverse primer):GCT TCC TGT AGG TGG CAA TC

Snail (Forward primer):TCG GAA GCC TAA CTA CAG CGA

Snail (Reverse primer):AGA TGA GCA TTG GCA GCG AG

**2. Details information of plasmid vectors and shRNAs used in gene transfection experiments.**

**Hes6 shNC & shRNA:**

GV493 (type)


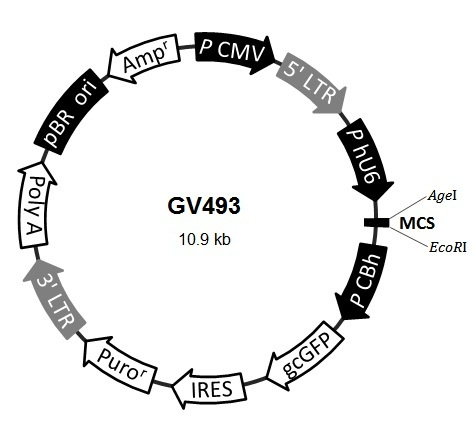


hU6-MCS-CBh-gcGFP-IRES-puromycinn (element sequences)

Target Seq of shRNA-1: CGAGCTCCTGAACCATCTGCT

Target Seq of shRNA-2: CGGCTACATCCAGTGCATGCA

Target Seq of shRNA-3: GCGGATCAACGAGAGCCTGCA

Target Seq of CON313(shNC): TTCTCCGAACGTGTCACGT

**Hes6 empty vector & Hes6:**

GV492 (type);

**
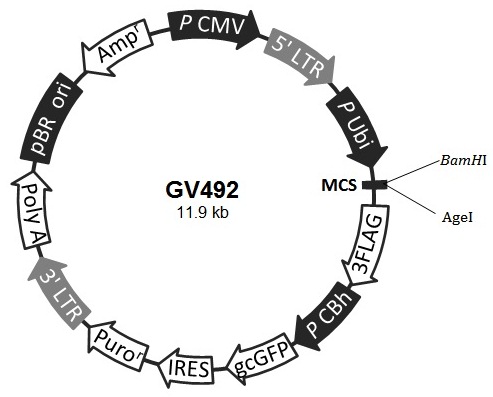
**

Ubi-MCS-3FLAG-CBh-gcGFP-IRES-puromycin (element sequences)

Target gene plasmid sequence ：

CTTGGGCTGCAGGTCGACTCTAGAGGATCCCGCCACCATGGCGCCACCCGCGGCGCCTGGCCGGGACCGTGTGGGCCGTGAGGATGAGGACGGCTGGGAGACGCGAGGGGACCGCAAGGCCCGGAAGCCCCTGGTGGAGAAGAAGCGGCGCGCGCGGATCAACGAGAGCCTGCAGGAGCTGCGGCTGCTGCTGGCGGGCGCCGAGGTGCAGGCCAAGCTGGAGAACGCCGAAGTGCTGGAGCTGACGGTGCGGCGGGTCCAGGGTGTGCTGCGGGGCCGGGCGCGCGAGCGCGAGCAGCTGCAGGCGGAAGCGAGCGAGCGCTTCGCTGCCGGCTACATCCAGTGCATGCACGAGGTGCACACGTTCGTGTCCACGTGCCAGGCCATCGACGCTACCGTCGCTGCCGAGCTCCTGAACCATCTGCTCGAGTCCATGCCGCTGCGTGAGGGCAGCAGCTTCCAGGATCTGCTGGGGGACGCCCTGGCGGGGCCACCTAGAGCCCCTGGACGGAGTGGCTGGCCTGCGGGGGGCGCTCCGGGATCCCCAATACCCAGCCCCCCGGGTCCTGGGGACGACCTGTGCTCCGACCTGGAGGAGGCCCCTGAGGCCGAACTGAGTCAGGCTCCTGCTGAGGGGCCCGACTTGGTGCCCGCAGCCCTGGGCAGCCTGACCACAGCCCAAATTGCCCGGAGTGTCTGGAGGCCTTGGACCGGTATGGACTACAAGGATGACGATGACAAGGATTACAAAGACGACGATGATAAGGACTATAAGGATGATGACGACAAATGAGCTAGCA

**3. Primary antibodies used in western blot, IF and IHC experiments.**

| **Antibody** | **Specices** | **Company** | **Serial number** |
| --- | --- | --- | --- |
| β-actin | mouse | HuaBio | EM21002 |
| Hes6 | rabbit | Immunoway | YT2127 |
| Hes6 | mouse | Abmart | MG280717 |
| Twist1 | rabbit | Proteintech | 25465-1-AP |
| E-cadherin | rabbit | Proteintech | 20874-1-AP |
| N-cadherin | rabbit | Abclonal | A0433 |
| Vimentin | rabbit | Proteintech | 10366-1-AP |
| Snail | rabbit | Abmart | TA6032 |
| PI3K(110α) | rabbit | CST | 4249S |
| PI3K(85α) | mouse | Proteintech | 60225-1-lg |
| Phospho-PI3K p85 | rabbit | Affinity | AF3242 |
| AKT | rabbit | CST | 4691T |
| Phospho-Akt | rabbit | CST | 4060T |
| Ki-67 | rabbit | HuaBio | HA721115 |
