# Supplementary material for: Transcription cofactor Hes6 interacts with Twist1 to facilitate EMT and promote gastric carcinogenesis by activating the PI3K/AKT signaling
Source: Genes Dis. 2025 May 5;13(5):101674. doi: 10.1016/j.gendis.2025.101674 (PMC13264254; doi:10.1016/j.gendis.2025.101674)
Supplement: Multimedia component 3 [file mmc3.docx]

**1. The main configuration parameters of fluorescence microscopy in this study.**

| **Properties** | **Details** |
| --- | --- |
| Device Type | Leica DM4B, Germany |
| Imaging Imodality | FlUO + IL |
| Camera | DFC7000T-0278754120 |
| Format | Bin1x1 (1920×1440) |
| Objective Lens Name | HC PL FLUOTAR 20×/0.55 DRY |
| Microscope Model | DM4B |
| Color Capture Mode | Composite |
| Quality Mode | 40 MHz |

**2.** **The main configuration parameters of laser confocal microscopy in this study.**

| **Properties** | **Details** |
| --- | --- |
| Device Type | Leica SP8, Germany |
| Pinhole Size | 95.6 μm |
| Scan Speed | 200 Hz |
| Magnification | 63 |
| Objective Lens Name | HC PL APO CS2 63×/1.40 OTL |
| **Lasers Parameters** | |
| Laser Name | Argon |
| Output Power | 19.84 % |
| Laser Line (405 nm) Intensity | 20.3 % |
| Laser Line (488 nm) Intensity | 7.00 % |
| Laser Line (561 nm) Intensity | 7.00 % |
| **Exposure Parameters** | |
| Exposure Time | < 100 ms |
| **Detectors** |  |
| Channel 1：PMT (415 nm - 472 nm) | Gain value：800 |
| Channel 2：PMT (498 nm - 563 nm) | Gain value：780.9 |
| Channel 3：PMT (604 nm - 677 nm) | Gain value：729.9 |
| **Filter Wheels/ Other Motorized Devices** | |
| **Device Name** | **Filter Name/Position** |
| Excitation Beam Splitter | TD 488/561/633 |
| Galvo Slider | Galvo X Normal |
| Galvo Resonant Pan | Galvo X Pan Center |
| Target Slider | Target Park |
| X2 Lens Changer | CS2 UV Optics 1 |

**3. Contact residues for binding between protein Hes6 and protein Twist1.**

| **Hes6** | **ΔGsolv*** | **Twist1** | **ΔGsolv*** |
| --- | --- | --- | --- |
| ARG 118 | -0.04132 | LEU 50 | 0.207866 |
| GLN 119 | -0.33518 | LEU 51 | 0.473569 |
| GLN 122 | -0.4377 | LEU 52 | -0.2136 |
| SER 123 | -0.32796 | ALA 53 | 0.323637 |
| LEU 124 | 0.414121 | GLY 54 | 0.506346 |
| GLU 126 | -0.23758 | ALA 55 | 0.125874 |
| ALA 127 | 0.868348 | GLU 56 | -0.00342 |
| PHE 128 | 0.248239 | VAL 57 | 0.890803 |
| ALA 129 | 0.155446 | GLN 58 | -0.03819 |
| ALA 130 | 0.79845 | LYS 60 | 0.145417 |
| LEU 131 | 0.645267 | LEU 61 | 0.005338 |
| LYS 133 | -1.28153 | GLU 65 | -0.01041 |
| ILE 134 | 0.95884 | LEU 69 | 0.663748 |
| LEU 149 | 0.877368 | ARG 72 | 0.060204 |
| ALA 152 | 0.09896 | ARG 73 | -1.23054 |
| ILE 156 | 0.394951 | GLY 76 | 0.110241 |
| - | - | VAL 77 | 0.068507 |
| - | - | LEU 203 | 0.073352 |
| - | - | VAL 204 | 0.007452 |
| - | - | PRO 205 | 0.984665 |
| - | - | ALA 207 | 0.181533 |
| - | - | LEU 208 | 0.809852 |

**4*.*** **Table of hydrogen bonding between Hes6 and Twist1 proteins.**

| **Hes6** | **Atomic name** | **Twist1** | **Atomic name** | **distance(Å)** |
| --- | --- | --- | --- | --- |
| LEU 131 | N | LEU 52 | O | 2.78357 |
| ALA 129 | O | ARG 73 | NH1 | 3.44574 |
